# Piperazine-1,4-diium bis­(2,4,5-tricarb­oxy­benzoate) dihydrate

**DOI:** 10.1107/S160053681300723X

**Published:** 2013-03-23

**Authors:** Nagaraju Narayanam, Kranthi Kumar Gangu, Balakrishna Kurra, Saratchandra Babu Mukkamala

**Affiliations:** a Department of Chemistry, GITAM University, Visakhapatnam 530 045, Andhra Pradesh, India

## Abstract

In the title hydrated salt, C_4_H_12_N_2_^2+^·2C_10_H_5_O_8_^−^·2H_2_O, the piperazinediium cation, lying about an inversion center, adopts a chair conformation. The benzene ring of the anion makes dihedral angles of 25.17 (8)° with the carboxyl­ate group and angles of 8.50 (7), 20.07 (7) and 80.86 (8)° with the three carb­oxy­lic acid groups. In the crystal, the cations, anions and water mol­ecules are connected by O—H⋯O and N—H⋯O hydrogen bonds into double layers parallel to (110).

## Related literature

For supra­molecular architectures involving benzene-1,2,4,5-tetra­carb­oxy­lic acid and its anions, see: Aghabozorg *et al.* (2006 ▶, 2008 ▶); Chiwei *et al.* (2005 ▶); Pasban *et al.* (2012 ▶); Pasdar *et al.* (2010 ▶); Smith *et al.* (2008 ▶); Smith & Wermuth (2010 ▶); Vaidhyanathan *et al.* (2002 ▶). For proton-transfer systems, see: Aghabozorg *et al.* (2010 ▶). For inter­molecular inter­actions, see: Janiak (2000 ▶).
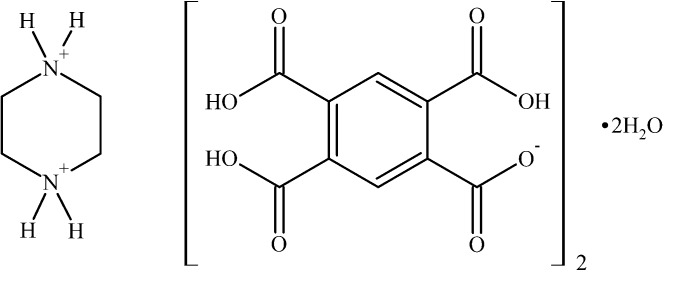


## Experimental

### 

#### Crystal data

C_4_H_12_N_2_^2+^·2C_10_H_5_O_8_^−^·2H_2_O
*M* *_r_* = 630.46
Triclinic, 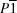

*a* = 8.2521 (2) Å
*b* = 8.4810 (2) Å
*c* = 9.6369 (2) Å
α = 87.117 (5)°
β = 89.527 (5)°
γ = 70.962 (4)°
*V* = 636.73 (3) Å^3^
*Z* = 1
Mo *K*α radiation
μ = 0.14 mm^−1^
*T* = 293 K
0.30 × 0.20 × 0.20 mm

#### Data collection

Bruker Kappa APEXII CCD diffractometer
Absorption correction: multi-scan (*SADABS*; Bruker, 2008 ▶) *T*_min_ = 0.945, *T*_max_ = 0.985
11108 measured reflections
2234 independent reflections
2061 reflections with *I* > 2σ(*I*)
*R*_int_ = 0.028

#### Refinement

*R*[*F* ^2^ > 2σ(*F* ^2^)] = 0.030
*wR*(*F* ^2^) = 0.085
*S* = 1.06
2234 reflections
224 parameters
4 restraints
H atoms treated by a mixture of independent and constrained refinement
Δρ_max_ = 0.21 e Å^−3^
Δρ_min_ = −0.20 e Å^−3^

### 

Data collection: *APEX2* (Bruker, 2008 ▶); cell refinement: *SAINT* (Bruker, 2008 ▶); data reduction: *SAINT*; program(s) used to solve structure: *SIR92* (Altomare *et al.*, 1993 ▶); program(s) used to refine structure: *SHELXL97* (Sheldrick, 2008 ▶); molecular graphics: *ORTEP-3 for Windows* (Farrugia, 2012 ▶) and *Mercury* (Macrae *et al.*, 2008 ▶); software used to prepare material for publication: *SHELXL97* and *DIAMOND* (Brandenburg, 2007 ▶).

## Supplementary Material

Crystal structure: contains datablock(s) I, global. DOI: 10.1107/S160053681300723X/yk2086sup1.cif

Structure factors: contains datablock(s) I. DOI: 10.1107/S160053681300723X/yk2086Isup2.hkl

Supplementary material file. DOI: 10.1107/S160053681300723X/yk2086Isup3.cml

Additional supplementary materials:  crystallographic information; 3D view; checkCIF report

## Figures and Tables

**Table 1:** Hydrogen-bond geometry (Å, °)

*D*—H⋯*A*	*D*—H	H⋯*A*	*D*⋯*A*	*D*—H⋯*A*
O1—H1⋯O8	0.82	1.63	2.4225 (15)	161
O3—H3⋯O7^i^	0.82	1.80	2.6100 (13)	167
O6—H6⋯O2^ii^	0.82	1.78	2.5884 (13)	170
N1—H1*A*⋯O9^iii^	0.90 (2)	1.83 (2)	2.7283 (17)	176 (2)
N1—H1*B*⋯O5^iv^	0.90 (2)	1.94 (2)	2.7420 (16)	147 (2)
O9—H9*A*⋯O8^v^	0.86 (2)	2.14 (2)	2.9904 (18)	174 (3)
O9—H9*B*⋯O7	0.82 (2)	2.18 (2)	2.9799 (19)	163 (3)

Symmetry codes: (i) 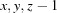; (ii) 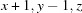; (iii) 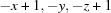; (iv) 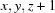; (v) 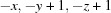.

**Fig. 1.**
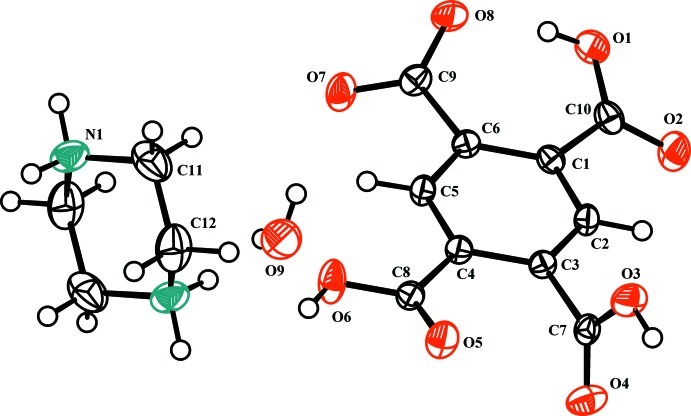
The view of the title compound showing the atom-labelling scheme. Displacement ellipsoids are drawn at the 50% probability level.

**Fig. 2.**
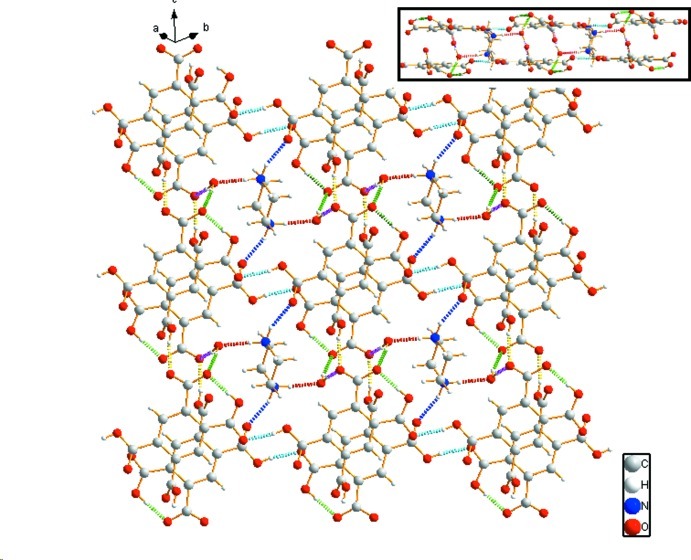
A perspective view of hydrogen bonding interactions represented by dashed lines. Insent: 2D supramolecular representation along c axis.

**Fig. 3.**
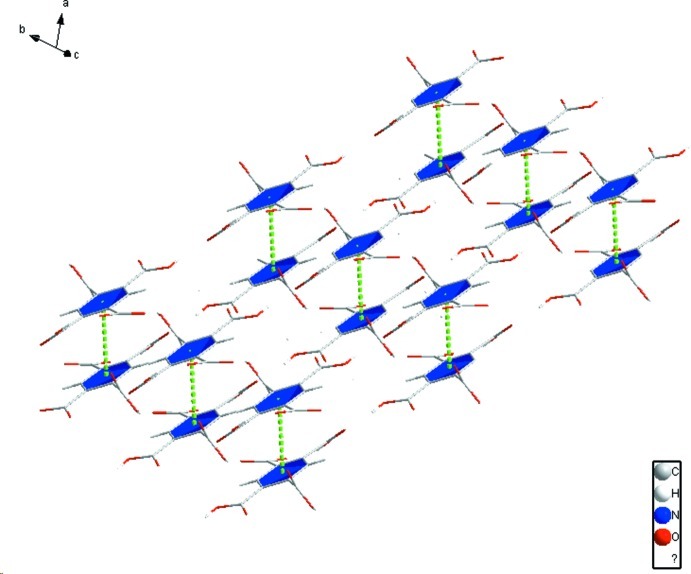
π-π stacking interactions between benzene-1,2,4,5-tetracarboxylates. Cations, water molecules and hydrogen atoms are omitted for clarity.

**Fig. 4.**
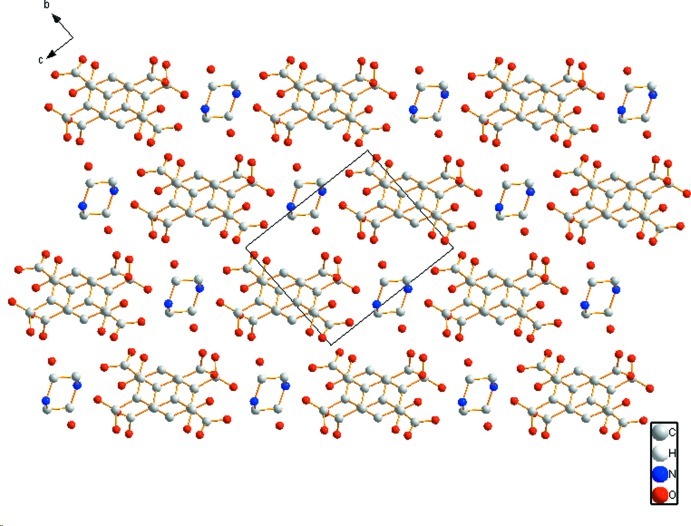
Crystal packing viewed along a axis. Hydrogen atoms are omitted for clarity.

## supplementary crystallographic information

### Comment

Benzene-1,2,4,5-tetracarboxylic acid (H4btc) with its ability to donate four protons is a versatile ligand in building supramolecular architectures and proton transfer compounds with nitrogen containing organic amines such as piperazine (Aghabozorg *et al.*, 2008; Vaidhyanathan *et al.*, 2002) and diethylenetriamine (Pasban *et al.*, 2012). A number of proton transfer compounds and supramolecular architectures were reported earlier with H4btc and other organic bases such as 1,10- phenanthroline (Chiwei *et al.*, 2005) and propane-1,2-diammonium (Pasdar *et al.*, 2010). These proton transfer compounds have the ability to absorb various metals into their crystal lattices (Aghabozorg *et al.*, 2006) with profound applications in metal separation and storage (Aghabozorg *et al.*, 2010; Smith *et al.*, 2008; Smith & Wermuth, 2010).

We report here one such proton transfer compound, piperazinediium bis(benzene-1,2,4,5-tetracarboxylate) dihydrate. As shown in Fig. 1, the asymmetric unit contain one mono-deprotonated residue of benzene-1,2,4,5-tetracaboxylic acid (H3btc^-^), a half of diprotonated piperazine (pipz) and one water molecule. Layered 2D supramolecular structure of title compound was built by connecting H3btc^-^, pipz and water molecules through hydrogen bonding (Fig.2). Inter- and intramolecular hydrogen bonding was observed between H3btc- molecular species through O–H···O hydrogen bonds. Piperazinediium cations and water molecules are involved in building this supramolecular structure through N–H···O and O–H···O bonds. Besides hydrogen bonding, the weak aromatic π-π stacking interactions between aromatic rings of H3btc- molecules could contribute for further stabilization of this layered supramolecular crystal structure. As shown in Fig. 3, the π-π stacking interactions between H3btc- (intercentroid separation of 3.7954 Å) were found to be in agreement with the reported values (Janiak, 2000). The packing diagram of the title compound is shown in Fig.4.

### Experimental

0.2974 g (1.0 mmol) of Zn(NO3)2.6H2O, 0.1270 g (0.5 mmol) of 1,2,4,5-benzenetetracarboxylic acid, 0.2583 g (3 mmol) of piperazine and 0.2 mL (4 mmol) of sulfuric acid were dissolved in 10.0 mL distilled water and heated in a stainless steel Teflon-lined autoclave at 120°C for 24 hours. The mixture was cooled to room temperature at a cooling rate of 6 °/min. Colorless cubic shaped crystals were obtained from the reaction mixture. Yield: 68% (based on H4btc). IR (KBr): 3473.27 (s), 3033.5 (w), 2548 (m), 1707 (s), 1621(s), 1475 (m), 734 (s), 596(s), 471(s).

### Refinement

Refinement on F2 against ALL reflections. The weighted R-factor wR2 and goodness of fit S were based on F2 and conventional R-factors R were based on F, with F set to zero for negative F2. The threshold expression of F2 > 2sig(F2) was used only for calculating R factors(gt) etc. and was not relevant to the choice of reflections for refinement. R-factors based on F2 were statistically about twice as large as those based on F and R- factors based on ALL data will be even larger.

### Crystal data

**Table:**

C_4_H_12_N_2_^2+^·2C_10_H_5_O_8_^−^·2H_2_O	*Z* = 1
*M**_r_* = 630.46	*F*(000) = 328
Triclinic, *P*1	*D*_x_ = 1.644 Mg m^−^^3^
Hall symbol: -P 1	Melting point: 560 K
*a* = 8.2521 (2) Å	Mo *K*α radiation, λ = 0.71073 Å
*b* = 8.4810 (2) Å	Cell parameters from 7490 reflections
*c* = 9.6369 (2) Å	θ = 2.5–33.0°
α = 87.117 (5)°	µ = 0.14 mm^−^^1^
β = 89.527 (5)°	*T* = 293 K
γ = 70.962 (4)°	Block, colourless
*V* = 636.73 (3) Å^3^	0.30 × 0.20 × 0.20 mm

### Data collection

**Table:**

Bruker Kappa APEXII CCD diffractometer	2234 independent reflections
Radiation source: fine-focus sealed tube	2061 reflections with *I* > 2σ(*I*)
Graphite monochromator	*R*_int_ = 0.028
ω and φ scan	θ_max_ = 25.0°, θ_min_ = 2.5°
Absorption correction: multi-scan (*SADABS*; Bruker, 2008)	*h* = −9→9
*T*_min_ = 0.945, *T*_max_ = 0.985	*k* = −9→10
11108 measured reflections	*l* = −11→11

### Refinement

**Table:**

Refinement on *F*^2^	Secondary atom site location: difference Fourier map
Least-squares matrix: full	Hydrogen site location: inferred from neighbouring sites
*R*[*F*^2^ > 2σ(*F*^2^)] = 0.030	H atoms treated by a mixture of independent and constrained refinement
*wR*(*F*^2^) = 0.085	*w* = 1/[σ^2^(*F*_o_^2^) + (0.0431*P*)^2^ + 0.2345*P*] where *P* = (*F*_o_^2^ + 2*F*_c_^2^)/3
*S* = 1.06	(Δ/σ)_max_ < 0.001
2234 reflections	Δρ_max_ = 0.21 e Å^−^^3^
224 parameters	Δρ_min_ = −0.20 e Å^−^^3^
4 restraints	Extinction correction: *SHELXL97* (Sheldrick, 2008), Fc^*^=kFc[1+0.001xFc^2^λ^3^/sin(2θ)]^-1/4^
Primary atom site location: structure-invariant direct methods	Extinction coefficient: 0.075 (5)

### Special details

**Table:**

Geometry. All e.s.d.'s (except the e.s.d. in the dihedral angle between two l.s. planes) are estimated using the full covariance matrix. The cell e.s.d.'s are taken into account individually in the estimation of e.s.d.'s in distances, angles and torsion angles; correlations between e.s.d.'s in cell parameters are only used when they are defined by crystal symmetry. An approximate (isotropic) treatment of cell e.s.d.'s is used for estimating e.s.d.'s involving l.s. planes.
Refinement. Refinement of *F*^2^ against ALL reflections. The weighted *R*-factor *wR* and goodness of fit *S* are based on *F*^2^, conventional *R*-factors *R* are based on *F*, with *F* set to zero for negative *F*^2^. The threshold expression of *F*^2^ > σ(*F*^2^) is used only for calculating *R*-factors(gt) *etc*. and is not relevant to the choice of reflections for refinement. *R*-factors based on *F*^2^ are statistically about twice as large as those based on *F*, and *R*- factors based on ALL data will be even larger.

### Fractional atomic coordinates and isotropic or equivalent isotropic displacement parameters (Å^2^)

**Table:**

	*x*	*y*	*z*	*U*_iso_*/*U*_eq_	
C1	0.06597 (16)	0.70913 (15)	0.05612 (13)	0.0202 (3)	
C2	0.08629 (16)	0.66166 (16)	−0.08067 (13)	0.0219 (3)	
H2	0.0098	0.7264	−0.1480	0.026*	
C3	0.21641 (16)	0.52142 (16)	−0.12023 (13)	0.0196 (3)	
C4	0.32899 (16)	0.42177 (15)	−0.01920 (13)	0.0198 (3)	
C5	0.30804 (16)	0.46709 (16)	0.11737 (13)	0.0212 (3)	
H5	0.3824	0.3995	0.1846	0.025*	
C6	0.18074 (16)	0.60918 (16)	0.15847 (13)	0.0200 (3)	
C7	0.22375 (16)	0.47981 (16)	−0.27035 (13)	0.0217 (3)	
C8	0.47247 (16)	0.27185 (16)	−0.05880 (13)	0.0217 (3)	
C9	0.18506 (17)	0.63946 (17)	0.31156 (14)	0.0241 (3)	
C10	−0.08355 (17)	0.86608 (16)	0.07812 (14)	0.0244 (3)	
C11	0.5751 (2)	0.1289 (2)	0.4758 (2)	0.0448 (4)	
H11A	0.6750	0.1621	0.4555	0.054*	
H11B	0.4771	0.2293	0.4836	0.054*	
C12	0.5451 (2)	0.0307 (2)	0.36090 (17)	0.0436 (4)	
N1	0.60207 (17)	0.02802 (17)	0.60844 (14)	0.0371 (3)	
O1	−0.08934 (13)	0.95103 (12)	0.18574 (11)	0.0337 (3)	
H1	−0.0039	0.9062	0.2342	0.051*	
O2	−0.19676 (13)	0.91343 (13)	−0.01098 (11)	0.0368 (3)	
O3	0.29608 (14)	0.56784 (13)	−0.34862 (10)	0.0327 (3)	
H3	0.2974	0.5411	−0.4293	0.049*	
O4	0.16086 (14)	0.38163 (14)	−0.31255 (10)	0.0348 (3)	
O5	0.50699 (13)	0.24168 (13)	−0.17891 (10)	0.0337 (3)	
O6	0.55721 (13)	0.18159 (13)	0.04674 (10)	0.0373 (3)	
H6	0.6341	0.1014	0.0187	0.056*	
O7	0.25414 (14)	0.51726 (13)	0.39115 (10)	0.0359 (3)	
O8	0.12443 (14)	0.78708 (13)	0.35313 (10)	0.0339 (3)	
O9	0.10687 (16)	0.23958 (16)	0.41278 (14)	0.0453 (3)	
H12B	0.646 (3)	−0.068 (3)	0.352 (2)	0.051 (5)*	
H12A	0.518 (3)	0.099 (3)	0.276 (2)	0.061 (6)*	
H1A	0.700 (2)	−0.060 (2)	0.605 (2)	0.046 (5)*	
H1B	0.615 (3)	0.092 (3)	0.677 (2)	0.064 (6)*	
H9A	0.043 (3)	0.224 (4)	0.479 (2)	0.097 (10)*	
H9B	0.135 (4)	0.323 (3)	0.421 (3)	0.106 (11)*	

### Atomic displacement parameters (Å^2^)

**Table:**

	*U*^11^	*U*^22^	*U*^33^	*U*^12^	*U*^13^	*U*^23^
C1	0.0199 (6)	0.0181 (6)	0.0217 (7)	−0.0048 (5)	0.0028 (5)	−0.0015 (5)
C2	0.0211 (6)	0.0216 (6)	0.0193 (6)	−0.0023 (5)	−0.0020 (5)	0.0016 (5)
C3	0.0203 (6)	0.0203 (6)	0.0177 (6)	−0.0061 (5)	0.0017 (5)	−0.0012 (5)
C4	0.0192 (6)	0.0200 (6)	0.0188 (6)	−0.0042 (5)	0.0012 (5)	−0.0016 (5)
C5	0.0216 (6)	0.0216 (6)	0.0172 (6)	−0.0029 (5)	−0.0011 (5)	0.0006 (5)
C6	0.0209 (6)	0.0211 (6)	0.0175 (6)	−0.0062 (5)	0.0021 (5)	−0.0021 (5)
C7	0.0194 (6)	0.0222 (7)	0.0192 (6)	−0.0010 (5)	−0.0010 (5)	−0.0009 (5)
C8	0.0211 (6)	0.0216 (7)	0.0204 (7)	−0.0039 (5)	−0.0003 (5)	−0.0029 (5)
C9	0.0229 (7)	0.0286 (7)	0.0200 (7)	−0.0067 (5)	0.0033 (5)	−0.0047 (5)
C10	0.0233 (7)	0.0205 (7)	0.0262 (7)	−0.0030 (5)	0.0047 (6)	−0.0002 (5)
C11	0.0417 (9)	0.0311 (8)	0.0642 (12)	−0.0163 (7)	−0.0010 (8)	0.0049 (8)
C12	0.0459 (10)	0.0509 (10)	0.0267 (8)	−0.0069 (8)	0.0089 (7)	0.0058 (7)
N1	0.0346 (7)	0.0356 (7)	0.0356 (7)	−0.0016 (6)	−0.0049 (6)	−0.0169 (6)
O1	0.0353 (6)	0.0245 (5)	0.0334 (6)	0.0025 (4)	0.0003 (4)	−0.0098 (4)
O2	0.0298 (6)	0.0326 (6)	0.0350 (6)	0.0084 (4)	−0.0040 (5)	−0.0059 (5)
O3	0.0481 (6)	0.0378 (6)	0.0168 (5)	−0.0202 (5)	0.0040 (4)	−0.0023 (4)
O4	0.0428 (6)	0.0422 (6)	0.0262 (5)	−0.0221 (5)	0.0022 (4)	−0.0100 (4)
O5	0.0354 (6)	0.0339 (6)	0.0210 (5)	0.0042 (4)	0.0023 (4)	−0.0086 (4)
O6	0.0347 (6)	0.0350 (6)	0.0230 (5)	0.0147 (4)	−0.0001 (4)	−0.0012 (4)
O7	0.0478 (7)	0.0342 (6)	0.0168 (5)	−0.0008 (5)	−0.0016 (4)	−0.0016 (4)
O8	0.0418 (6)	0.0303 (6)	0.0258 (5)	−0.0049 (5)	−0.0001 (4)	−0.0127 (4)
O9	0.0401 (7)	0.0411 (7)	0.0463 (8)	−0.0006 (6)	−0.0027 (6)	−0.0112 (6)

### Geometric parameters (Å, º)

**Table:**

C1—C2	1.3906 (18)	C10—O2	1.2269 (17)
C1—C6	1.4127 (18)	C10—O1	1.2841 (17)
C1—C10	1.5138 (17)	C11—N1	1.479 (2)
C2—C3	1.3851 (18)	C11—C12	1.487 (3)
C2—H2	0.9300	C11—H11A	0.9700
C3—C4	1.3947 (18)	C11—H11B	0.9700
C3—C7	1.5024 (17)	C12—N1^i^	1.478 (2)
C4—C5	1.3833 (18)	C12—H12B	0.98 (2)
C4—C8	1.4911 (17)	C12—H12A	0.96 (2)
C5—C6	1.3885 (18)	N1—C12^i^	1.478 (2)
C5—H5	0.9300	N1—H1A	0.904 (15)
C6—C9	1.5129 (18)	N1—H1B	0.901 (16)
C7—O4	1.2031 (17)	O1—H1	0.8200
C7—O3	1.3074 (16)	O3—H3	0.8200
C8—O5	1.2096 (16)	O6—H6	0.8200
C8—O6	1.3014 (16)	O9—H9A	0.858 (17)
C9—O7	1.2367 (17)	O9—H9B	0.824 (18)
C9—O8	1.2715 (17)		
			
C2—C1—C6	118.71 (11)	O8—C9—C6	119.92 (12)
C2—C1—C10	114.30 (11)	O2—C10—O1	120.53 (12)
C6—C1—C10	126.99 (12)	O2—C10—C1	118.69 (12)
C3—C2—C1	122.32 (12)	O1—C10—C1	120.74 (12)
C3—C2—H2	118.8	N1—C11—C12	110.21 (13)
C1—C2—H2	118.8	N1—C11—H11A	109.6
C2—C3—C4	119.08 (12)	C12—C11—H11A	109.6
C2—C3—C7	117.76 (11)	N1—C11—H11B	109.6
C4—C3—C7	123.09 (11)	C12—C11—H11B	109.6
C5—C4—C3	118.88 (11)	H11A—C11—H11B	108.1
C5—C4—C8	120.72 (11)	N1^i^—C12—C11	110.90 (13)
C3—C4—C8	120.38 (11)	N1^i^—C12—H12B	107.1 (11)
C4—C5—C6	122.85 (12)	C11—C12—H12B	109.1 (12)
C4—C5—H5	118.6	N1^i^—C12—H12A	106.8 (12)
C6—C5—H5	118.6	C11—C12—H12A	110.4 (13)
C5—C6—C1	118.16 (11)	H12B—C12—H12A	112.5 (17)
C5—C6—C9	114.29 (11)	C12^i^—N1—C11	110.89 (13)
C1—C6—C9	127.54 (11)	C12^i^—N1—H1A	110.4 (12)
O4—C7—O3	124.71 (12)	C11—N1—H1A	110.1 (12)
O4—C7—C3	122.38 (12)	C12^i^—N1—H1B	109.9 (14)
O3—C7—C3	112.81 (11)	C11—N1—H1B	108.3 (14)
O5—C8—O6	124.18 (12)	H1A—N1—H1B	107.1 (19)
O5—C8—C4	121.94 (12)	C10—O1—H1	109.5
O6—C8—C4	113.86 (11)	C7—O3—H3	109.5
O7—C9—O8	122.61 (12)	C8—O6—H6	109.5
O7—C9—C6	117.42 (11)	H9A—O9—H9B	112 (3)
			
C6—C1—C2—C3	−0.96 (19)	C4—C3—C7—O4	81.46 (17)
C10—C1—C2—C3	179.89 (12)	C2—C3—C7—O3	80.87 (15)
C1—C2—C3—C4	1.5 (2)	C4—C3—C7—O3	−102.12 (14)
C1—C2—C3—C7	178.63 (12)	C5—C4—C8—O5	−170.29 (13)
C2—C3—C4—C5	−0.59 (19)	C3—C4—C8—O5	7.9 (2)
C7—C3—C4—C5	−177.56 (12)	C5—C4—C8—O6	8.07 (18)
C2—C3—C4—C8	−178.79 (11)	C3—C4—C8—O6	−173.75 (12)
C7—C3—C4—C8	4.23 (19)	C5—C6—C9—O7	−24.00 (18)
C3—C4—C5—C6	−0.9 (2)	C1—C6—C9—O7	157.18 (13)
C8—C4—C5—C6	177.35 (12)	C5—C6—C9—O8	153.59 (13)
C4—C5—C6—C1	1.4 (2)	C1—C6—C9—O8	−25.2 (2)
C4—C5—C6—C9	−177.56 (12)	C2—C1—C10—O2	18.28 (18)
C2—C1—C6—C5	−0.46 (18)	C6—C1—C10—O2	−160.79 (13)
C10—C1—C6—C5	178.57 (12)	C2—C1—C10—O1	−159.58 (12)
C2—C1—C6—C9	178.31 (12)	C6—C1—C10—O1	21.4 (2)
C10—C1—C6—C9	−2.7 (2)	N1—C11—C12—N1^i^	56.82 (19)
C2—C3—C7—O4	−95.55 (16)	C12—C11—N1—C12^i^	−56.81 (19)

Symmetry code: (i) −*x*+1, −*y*, −*z*+1.

### Hydrogen-bond geometry (Å, º)

**Table:**

*D*—H···*A*	*D*—H	H···*A*	*D*···*A*	*D*—H···*A*
O1—H1···O8	0.82	1.63	2.4225 (15)	161
O3—H3···O7^ii^	0.82	1.80	2.6100 (13)	167
O6—H6···O2^iii^	0.82	1.78	2.5884 (13)	170
N1—H1*A*···O9^i^	0.90 (2)	1.83 (2)	2.7283 (17)	176 (2)
N1—H1*B*···O5^iv^	0.90 (2)	1.94 (2)	2.7420 (16)	147 (2)
O9—H9*A*···O8^v^	0.86 (2)	2.14 (2)	2.9904 (18)	174 (3)
O9—H9*B*···O7	0.82 (2)	2.18 (2)	2.9799 (19)	163 (3)

Symmetry codes: (i) −*x*+1, −*y*, −*z*+1; (ii) *x*, *y*, *z*−1; (iii) *x*+1, *y*−1, *z*; (iv) *x*, *y*, *z*+1; (v) −*x*, −*y*+1, −*z*+1.
